# Aqueous Extract of the Edible *Gracilaria tenuistipitata* Inhibits Hepatitis C Viral Replication via Cyclooxygenase-2 Suppression and Reduces Virus-Induced Inflammation

**DOI:** 10.1371/journal.pone.0057704

**Published:** 2013-02-28

**Authors:** Kuan-Jen Chen, Chin-Kai Tseng, Fang-Rong Chang, Jin-Iong Yang, Chi-Chen Yeh, Wei-Chun Chen, Shou-Fang Wu, Hsueh-Wei Chang, Jin-Ching Lee

**Affiliations:** 1 Department of Biotechnology, College of Life Science, Kaohsiung Medical University, Kaohsiung, Taiwan; 2 Graduate Institute of Natural Products, College of Pharmacy, Kaohsiung Medical University, Kaohsiung, Taiwan; 3 Department of Seafood Science, National Kaohsiung Marine University, Kaohsiung, Taiwan; 4 Department of Biomedical Science and Environmental Biology, Cancer Center, Kaohsiung Medical University Hospital, Kaohsiung Medical University, Kaohsiung, Taiwan; University of Modena & Reggio Emilia, Italy

## Abstract

Hepatitis C virus (HCV) is an important human pathogen leading to hepatocellular carcinoma. Using an *in vitro* cell-based HCV replicon and JFH-1 infection system, we demonstrated that an aqueous extract of the seaweed *Gracilaria tenuistipitata* (AEGT) concentration-dependently inhibited HCV replication at nontoxic concentrations. AEGT synergistically enhanced interferon-α (IFN-α) anti-HCV activity in a combination treatment. We found that AEGT also significantly suppressed virus-induced cyclooxygenase-2 (COX-2) expression at promoter transactivation and protein levels. Notably, addition of exogenous COX-2 expression in AEGT-treated HCV replicon cells gradually abolished AEGT anti-HCV activity, suggesting that COX-2 down-regulation was responsible for AEGT antiviral effects. Furthermore, we highlighted the inhibitory effect of AEGT in HCV-induced pro-inflammatory gene expression such as the expression of tumour necrosis factor-α, interleukin-1β, inducible nitrite oxide synthase and COX-2 in a concentration-dependent manner to evaluate the potential therapeutic supplement in the management of patients with chronic HCV infections.

## Introduction

Hepatitis C virus (HCV) infection is the primary etiological agent of chronic hepatitis, liver cirrhosis and eventually hepatocellular carcinoma (HCC) [1], [2]. Till date, no prophylactic vaccine is available for HCV infection, and current therapy is primarily restricted to a combination of pegylated interferon-α (IFN-α) and ribavirin [3], [4]. However, IFN-based therapy is fraught with severe side effects and has various sustained virological response (SVR) rates, which are depended on the HCV genotype [5]. Despite the recent advancement of therapeutics with the approval of NS3/4A protease inhibitors, telaprevir and boceprevir, in combination with pegylated-interferon alpha (IFN-α) and ribavirin, the emergence of drug-resistant variants and the unfavourable side effects, such as anaemia and rash, may reduce the susceptibility and applicability of current HCV triple therapies [6]. Therefore, it is essential to develop alternate drugs or supplements for treating HCV infections.

HCV belongs to the *Flaviviridae* family and has a 9.6-kb single-stranded positive-sense RNA genome encoding a single precursor polyprotein of approximately 3000 amino acids, which generate 10 individual proteins, including structural proteins (core, E1, and E2) and non-structural (NS2, NS3, NS4A, NS5A and NS5B) proteins, processed by both host and viral proteases [7]. Among the latter, NS5A is a serine phosphoprotein, which exists as an important risk factor for hepatocarcinogenesis as a transcriptional activator of chronic inflammation by stimulating crucial mediators such as nuclear transcription factor-kappaB (NF-κB) and cyclooxygenase-2 (COX-2) [8], [9], [10]. COX-2, in particular, is an inducible Cox isozyme, and COX-2 overexpression is closely associated with human tumour formation by the production of large amounts of various prostaglandins (PGs), such as prostaglandin E2, thromboxane B2 and prostacyclin, which in turn cause the promotion of cellular proliferation, cancer invasiveness, angiogenesis and anti-apoptosis [11], [12]. Therefore, the inhibition of improper COX-2 expression is now considered to be a potential approach for cancer prevention and treatment [13]. In addition to developing a promising anti-tumour strategy by targeting the COX-2 signalling pathway, increasing evidence, including our previous studies, links the inhibition of COX-2 expression to anti-HCV activity at present [14], [15], [16],[17]. Therefore, an attractive means of eliminating HCV-related HCC is by combining anti-HCV and anti-inflammatory agents.

Many species of marine algae comprise a rich source of active substances, such as diterpenes and sulfated polysaccharides, which are potentially used for treating infectious diseases [18], [19]. *Gracilaria,* a genus of red algae, is abundantly produced in Taiwan. Numerous *Gracilaria* extracts promote various bioactivities including anti-oxidation [20], [21], [22], anti-hypercholesterolemia, anti-inflammation [23] and anti-microbial activities [22], [24]. More recently, our results indicated that the aqueous extracts of the *Gracilaria tenuistipitata* (AEGT) exhibited anti-oxidant activity and protective effect on H_2_O_2_-induced DNA damage [25]. In contrast, ethanolic and methanolic extracts of *G. tenuistipitata* exhibited anti-proliferation and induction of apoptosis on oral cancer cells [26], [27]. The effect of *Gracilaria* algae on human viral infection has not been investigated till date. The objectives of the present study were to evaluate the inhibitory effect of AEGT on HCV replication and to investigate the molecular mechanism underlying the anti-viral activity of AEGT by evaluating its inhibitory effect on COX-2 expression and upstream transcription factor, NF-κB. We subsequently examined whether the combination of AEGT and IFN-α produced synergistic effects that could inhibit HCV replication. Furthermore, we highlighted the inhibitory effect of AEGT on HCV-induced inflammatory mediators including tumour necrosis factor-α (TNF-α), interleukin-1β (IL-1β), inducible nitric oxide synthase (iNOS) and COX-2 to assess its potential supplement use against HCV-related HCC.

## Materials and Methods

### Ethics Statement

*G. tenuistipitata* is not an endangered or protected seaweed. *G. tenuistipitata* was collected in the No.129, Kouhu village, Kouhu Township, Yunlin County 653, Taiwan (R.O.C.), which is not a protected area but is privately owned by SHUI-TUI LI Taiwan. Mr. LI permits research in the reserves, and no specific permits are required for field studies such as the one described here.

### Preparation of Aqueous Extract of *G. tenuistipitata* (AEGT)

Raw materials. *G. tenuistipitata* was collected in the spring of 2009 from a culture farm at Kouhu beach, Yunlin County, Taiwan and delivered to our laboratory at 0°C. In the laboratory, the seaweeds were washed with tap water to remove epiphytes and encrusting material, soaked in distilled water twice and then lyophilised. The dried samples were pulverized, passed through a 60-mesh sieve, ground to a fine powder and stored at −40°C.

### Analysis of AEGT Extract: Polyphenol, Flavonoid, and Ascorbic Acid

Determination of bioactive components of the AEGT extract was performed as described previously [25], which included polyphenol, flavonoid, and ascorbic acid. The amount of polyphenol, flavonoid, and ascorbic acid were respectively recorded as 98.94±2.43 µg, 22.59±1.08 µg, and 1.59±0.18 µg, respectively, in 1 mg of dry extract.

### Cell Culture

Huh-7 cells were maintained in Dulbecco’s modified Eagle’s medium (DMEM) with 10% heat-inactivated foetal bovine serum (FBS), 1% antibiotic–antimycotic solution and 1% nonessential amino acids and incubated at 37°C with a 5% CO_2_ supplement. Ava5 cells (Huh-7 cells containing the subgenomic HCV genotype 1b replicon) were cultured in DMEM with 10% heat-inactivated FBS, 1% antibiotic–antimycotic solution, 1% nonessential amino acids and 1 mg/ml G418 and were incubated at 37°C with a 5% CO_2_ supplement.

### Western Blotting Assay

Western blotting was performed as described previously [17]. In brief, membrane samples were probed with monoclonal antibody specific for HCV NS5B (1∶5000; Abcam, Cambridge, MA), glyceraldehyde-3-phosphate dehydrogenase (GAPDH) (1∶10,000; GeneTex, Irvine, CA), c-Myc (1∶1000; GeneTex, Irvine, CA) or COX-2 (1∶1000; Cayman, Ann Arbor, MI) and NF-κB p65 subunit (1∶2000; GeneTex) or lamin B (1;1000; GeneTex). Signals were visualized by using the ECL Detection Kit (PerkinElmer, CT).

### Quantitative Real-Time Polymerase Chain Reaction (PCR)

HCV or cellular RNA was measured by quantitative real-time reverse-transcription PCR (qRT-PCR) analysis as described previously [17]. The levels of HCV NS5B, TNF-α, IL-1β, iNOS and COX-2 RNA were detected by qRT-PCR with the following forward and reverse primer sets: NS5B, 5′-GGA AAC CAA GCT GCC CAT CA-3′ and 5′-CCT CCA CGG ATA GAA GTT TA-3′; TNF-α, 5′-CCT GTG AGG AGG ACG AAC-3′ and 5′-AAG TGG TGG TCT TGT TGC-3′; IL-1β, 5′-GGA GAA TGA CCT GAG CAC-3′ and 5′-GAC CAG ACA TCA CCA AGC-3′; iNOS, 5′-CTT TGG TGC TGT ATT TCC-3′ and 5′-TGT GAC CTC AGA TAA TGC-3′ and COX-2, 5′-CCG AGG TGT ATG TAT GAG-3′ and 5′-TGG GTA AGT ATG TAG TGC-3′. The relative RNA level of these genes in each sample was normalized to cellular *gapdh* mRNA with the forward primer 5′-GTC TTC ACC ACC ATG GAG AA-3′ and reverse primer 5′-ATG GCA TGG ACT GTG GTC AT-3′.

### Cytotoxicity Assay

Cell viability was determined by the colorimetric 3-(4,5-dimethylthiazol-2-yl)-5- (3-carboxymethoxyphenyl)-2-(4-sulfophenyl)-2H-tetrazolium (MTS) assay (Promega Corporation, Madison, WI) as described previously [17]. Ava5 cells were seeded in 96-well plates at a density of 5×10^3^ per well and treated with AEGT at different concentrations. After 3 days of incubation, the medium was replaced by an MTS mixture containing 100 µl of phenol red-free medium and 20 µl of MTS reagent for 4 h at 37°C. Following this, absorbances were measured using a microplate reader at 490 nm.

### HCV JFH-1 Infection Assay

Production of infectious HCV genotype 2a JFH-1 particles and titration of generated HCV were performed as described [28]. By transfection of *in vitro-*transcribed full-length JFH-1 RNA into Huh-7.5, the infectious viral particles were generated. The inhibitory effect of AGET on HCV infection was assayed as described previously [29]. In brief, the Huh-7 cells were seeded at density of 4×10^4^ cells per well in a 24-wells culture plate and infected with 100 µl of HCV JFH-1 particles at a multiplicity of infection (MOI) of 0.1 for 6 h followed by incubation with various concentrations of AEGT for an additional 72 h. Subsequently, total RNA was collected and subjected to RT-qPCR for measuring mRNAs of HCV and GAPDH as described above.

### Synergy Analysis: Synergistic Isobologram

Ava5 cells were incubated with AEGT (0, 75, 150, 300 and 600 µg/ml) in combination with IFN-α (0, 7.5, 15, 30 and 60 U/ml) for 3 days. The inhibition of multiple drug dose effects were analysed using the CalcuSyn software (Biosoft, Cambridge, UK) based on the method of Chou and Talalay [30]. Calculated combination index (CI) was used to differentiate the presence of synergism (CI <1), additivity (CI  = 1) and antagonism (CI >1).

### Transient Transfection and Luciferase Activity Assay

Ava5 cells were seeded in 24-well plates at a density of 5×10^4^ per well and were transfected with 1 µg of plasmid pCOX-2-Luc or pNF-κB-Luc (BD Biosciences Clontech, Palo Alto, CA) using T-Pro™ reagent (Ji-Feng Biotechnology Co. Ltd., Taiwan) in accordance with the manufacturer’s instructions. Six hours post-transfection, the cells were treated with various concentrations of AEGT for 3 days. To further investigate COX-2 regulation by AEGT, Ava5 cells were transfected with serially increased concentrations of COX-2 expression vector (pCMV-COX-2-Myc) from 0.5 to 2 µg, and then treated with 600 µg/ml of AEGT for 3 days. The luciferase activity of cell extracts from each sample was measured using the Bright-Glo™ Luciferase Assay System (Promega) according to the manufacturer’s protocol. To determine the relationship between AEGT and NS5A-induced inflammatory genes (TNF-α, IL-1β, iNOS and COX-2), Huh-7 cells were transfected with 1 µg of plasmid pCMV-NS5A-Myc in the presence of AEGT (200 and 800 µg/ml) for 3 days. Subsequently, total RNA was subjected to qRT-PCR with specific primers.

### Intracellular Prostaglandin E2 (PGE_2_) Measurements

Ava5 cells were treated with AEGT at various concentrations for 3 days. Following drug treatment, cultured cells were thoroughly washed with cold phosphate-buffered saline (pH 7.4), and cell membranes were ruptured using lysis reagent containing C_15_H_34_BrN to release intracellular PGE_2_. The levels of PGE_2_ levels were then analyzed by PGE_2_ enzyme-linked immunosorbent assay system (Biotrak, Amersham Bioscience) according to the manufacturer’s protocol.

### Preparation of Nuclear Fraction

Nuclear extracts were prepared using NE-PER Nuclear and Cytoplasmic Extraction Reagents (Thermo Fisher Scientific Inc., USA) according to the manufacturer’s instructions. In brief, approximately 1×10^6^ Ava5 cells were seeded onto 10-cm dish for 24 h and then treated with or without AEGT. After 4 days of incubation, the cells were homogenated in hypotonic buffer (10 mM HEPES, 1 mM MgCl_2_, 1 mM EDTA, 10 mM KCl, 0.5 mM DTT, 0.5% Nonidet P-40, 4 µg/ml leupeptin, 20 µg/ml aprotinin, and 0.2 mM PMSF), followed by centrifugation at 2000 g for 5 min. Nuclear extracts were prepared using the hypotonic buffer after centrifugation at 7000 g for 15 min, following which the pellets containing crude nuclei were resuspended in the hypertonic buffer at 4°C for 30 min. After centrifugation at 20000 g for 15 min, nuclear proteins were collected and stored at −80°C until use.

### Statistical Analysis

The data were expressed as mean ± SD of at least three independent experiments. Statistical calculations were analysed by the Student’s *t*-test; *p*-values <0.01 were considered statistically significant.

## Results

### AEGT Inhibits HCV Protein Synthesis, RNA Replication and HCV Infection

**Figure 1:**
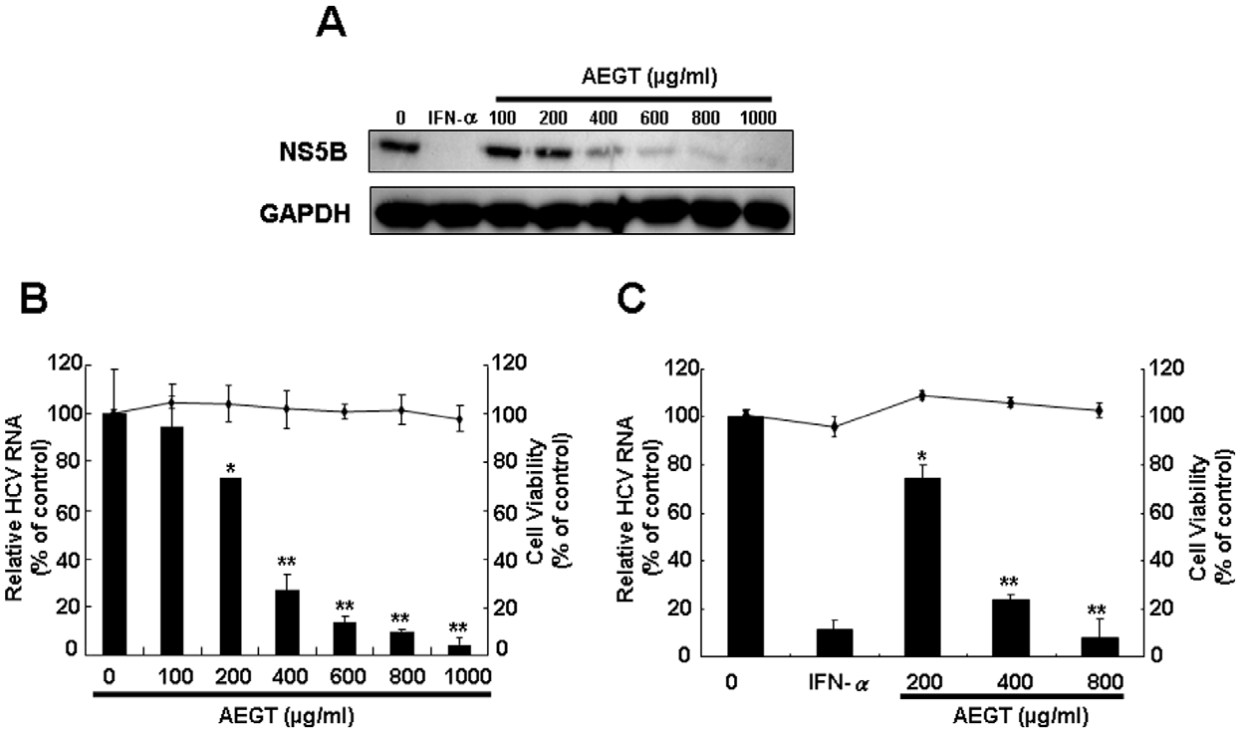
Inhibitory effect of AEGT on HCV protein synthesis, RNA replication and HCV infection. (A) Concentration-dependent reduction of HCV NS5B protein levels in AEGT-treated HCV replicon cells. Ava5 cells were treated with AEGT for 3 days at the indicated concentrations. Treatment with 100 U/ml IFN-α served as a positive control. Equal amounts of protein extracts were subjected to Western blotting with anti-NS5B and anti-GAPDH (a loading control) antibodies. (B) Concentration-dependent reduction of HCV RNA replication in AEGT-treated HCV replicon cells. HCV RNA levels were quantified by qRT-PCR and normalized to cellular *gapdh* mRNA levels following AEGT treatment for 3 days. Cellular toxicity was evaluated by the MTS assay after 3 days of incubation with AEGT. (C) Concentration-dependent reduction of infectious HCV JFH-1 replication in AEGT-treated Huh-7 cells. After 6 h of JFH-1 infection, Huh7-infected cells were treated with AEGT for 3 days. The levels of intracellular HCV RNA were determined by qRT-PCR following normalization of cellular *gapdh* mRNA. Results are represented as the mean ± SD (error bar) of three independent experiments. *P<0.05; ** *P*<0.01.

Initially, we assessed the effect of AEGT on HCV protein synthesis in Ava5 cells harboring an HCV subgenomic replicon [31]. HCV protein levels were determined by Western blotting using anti-HCV NS5B antibody. We found that different concentrations (100, 200, 400, 600, 800 and 1000 µg/ml) of AEGT markedly inhibited HCV protein synthesis in a concentration-dependent manner 3 days after treatment (Fig. 1A). To verify this finding, we next analyzed the effects of AEGT on HCV RNA replication using qRT-PCR analysis. Consistent with the results of the inhibitory effects of AEGT on viral protein synthesis, AEGT was also found to inhibit HCV RNA replication in a concentration-dependent manner, with an EC_50_ value of 300±0.3 µg/ml, as normalized by cellular *gapdh* mRNA (Fig. 1B). To rule out the possibility that AEGT anti-viral activity was caused by cytotoxic effects, cell proliferation was analyzed using the MTS assay. As shown in Fig. 1B, no significant cytotoxicity was detected at high AEGT concentrations (up to 1000 µg/ml). Using an HCV JFH-1 infection system [32], we confirmed the anti-HCV activity of AEGT, with an EC_50_ value of 325±0.7 µg/ml (Fig. 1C).

### Combination of IFN-α and AEGT Synergistically Inhibits HCV Replication

Next, we examined the anti-HCV of AEGT in combination with IFN-α. Ava5 cells were incubated with a combination of fixed concentrations of AEGT and IFN-α. HCV RNA replication inhibition was detected by qRT-PCR analysis. Based on the results of CalcuSyn™ analysis, a combination of IFN-α and AEGT at concentrations of 1∶5, 1∶10 and 1∶20 was found to have synergistic anti-HCV effects, as revealed by the combination index (CI) values of <1 for ED_50_, ED_75_ and ED_90_ (range, 0.89–0.52) (Table 1). These results indicated that AEGT may be a promising adjuvant for combination HCV therapy.

**Table 1:** Combined AEGT and IFN-α inhibitory effects on HCV RNA replication.

IFN-α:AEGT	CI Values at			Influence
	ED50	ED75	ED90	
1∶5	0.86	0.79	0.73	Synergistic
1∶10	0.85	0.82	0.78	Synergistic
1∶20	0.52	0.67	0.89	Synergistic

Ava5 cells were treated with combinations of various concentrations of AEGT and IFN-α (1∶5, 1∶10 or 1∶20) for 3 days. Anti-HCV activity was reflected by the reduction of HCV RNA level using qRT-PCR analysis. Mutual influence between AEGT and IFN-α was calculated using the CalcuSyn™ software, which produces the combination index (CI) values for various effective doses of 50% (ED_50_), 75% (ED_75_) and 90% (ED_90_). CI values indicate the degree of interaction of potential drugs; values of <1, 1 and >1 indicate synergistic, additive, or antagonistic effect, respectively.

### Anti-HCV Activity of AEGT Involves COX-2 and NF-κB Suppression

**Figure 2:**
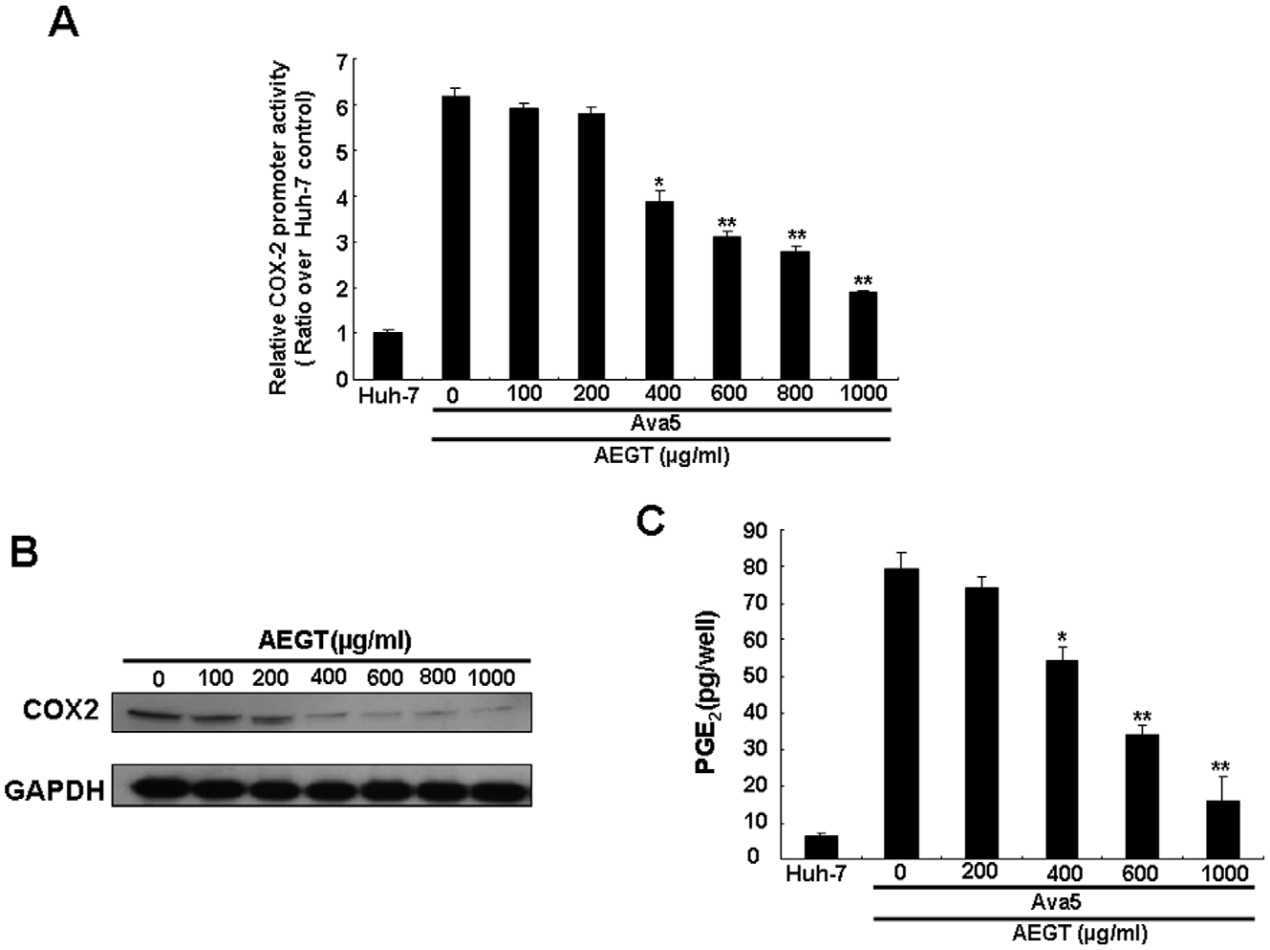
AEGT inhibitory effect on HCV-induced COX-2 promoter-linked reporter, protein expression and enzymatic activity. (A) Concentration-dependent reduction of COX-2 promoter-linked luciferase activity in AEGT-treated HCV replicon cells. Ava5 cells were transfected with the reporter plasmid pCOX-2-Luc and treated with indicated concentrations of AEGT for 3 days. Cell lysates were prepared for luminescence detection using the Steady-Glo Luciferase Assay Kit (Promega). The Huh-7 cells, transfected with pCOX-2-Luc without AEGT treatment, served as the basal level of COX-2 promoter activity, which is defined as 1. (B) Concentration-dependent COX-2 reduction of AEGT-treated HCV replicon cells. Ava5 cells were treated with AEGT for 3 days at the indicated concentrations. Cell lysates were prepared, and equal amounts of protein extracts (40 µg) were subjected to Western blot analysis with anti-COX-2 and anti-GAPDH (a loading control) antibodies. (C) AEGT inhibitory effect on PGE_2_ production. After 3 days of treatment, the intercellular PGE_2_ levels were analysed using the Biotrak PGE_2_ enzyme immunoassay system (Amersham). Non-treated Huh-7 cells served as the control. Each value represents the mean ± SD of three independent experiments. *P<0.05; ** *P*<0.01.

**Figure 3:**
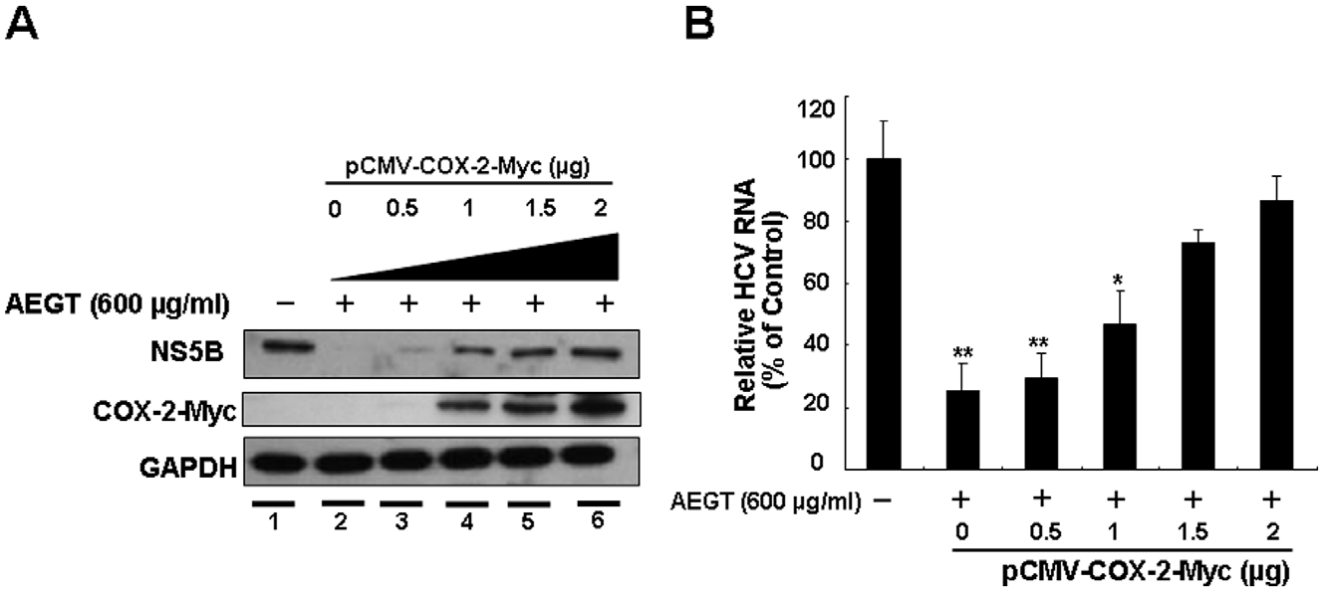
Involvement of COX-2 reduction in anti-HCV activity of AEGT. Concentration-dependent restoration of (A) HCV protein synthesis and (B) RNA replication by extraneous COX-2 expression in AEGT-treated Ava5 cells. Ava5 cells were transfected with increasing amounts (0.5–2 µg) of the plasmid pCMV-COX-2-Myc in the presence of 600 µg AEGT for 3 days. Total cell lysates were subjected to Western blotting with anti-NS5B, anti-Myc and anti-GAPDH (a loading control) antibodies. The HCV RNA levels were quantified by qRT-PCR following normalization of cellular *gapdh* mRNA levels. Each value was represented as the mean ± SD of three independent experiments. *P<0.05; ** *P*<0.01.

**Figure 4:**
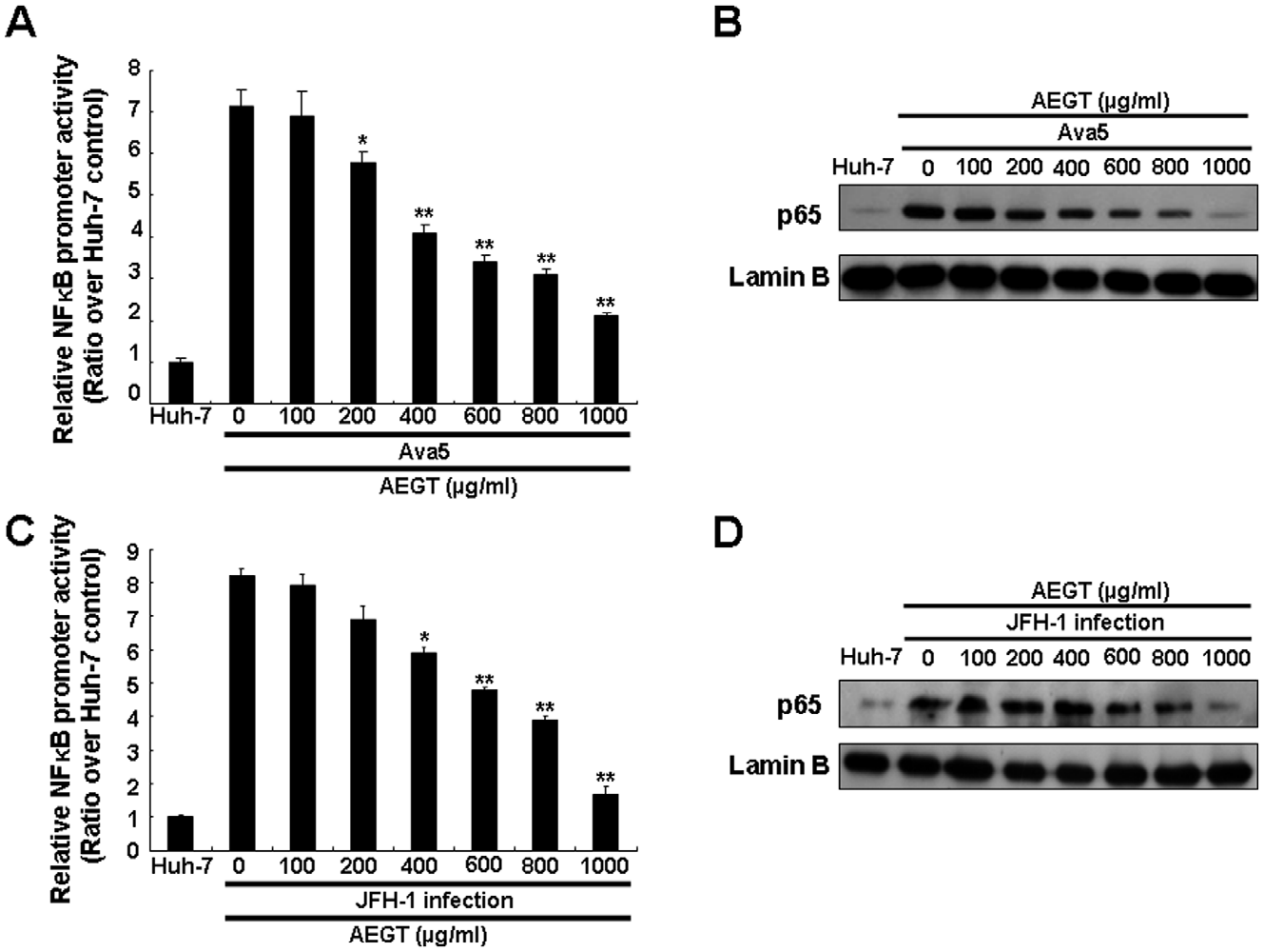
AEGT suppression of HCV-induced NF-κB activation. (A) Concentration-dependent reduction of NF-κB promoter-linked luciferase activity in AEGT-treated HCV replicon cells. Ava5 cells were transfected with the reporter plasmid pNF-κB-Luc in the presence of AEGT at indicated concentrations for 3 days. Total cell lysates were prepared for luminescence detection using the Steady-Glo Luciferase Assay Kit (Promega). Non-treated Huh-7 cells served as the basal control, which is defined as 1. (B) Concentration-dependent reduction of nuclear p65 protein levels in AEGT-treated HCV replicon cells. Nuclear extracts were prepared from AEGT-treated Ava5 cells and subjected to Western blot analysis using anti-NF-κB p65 and laminB antibodies, in which lamin B was used as a nuclear fraction control. (C) Concentration-dependent reduction of NF-κB promoter-linked luciferase activity by AEGT in JFH-1-infected Huh-7 cells. After 6 h of JFH-1 infection, Huh-7-infected cells were treated with AEGT at indicated concentrations for 3 days. Non-infected Huh-7 cells served as the basal control, which is defined as 1. Luciferase activity assay was performed as described above. (D) Concentration-dependent reduction of nuclear p65 protein levels by AEGT in JFH-1-infected Huh-7 cells. Western blot analysis was performed as described above. Each value was represented as the mean ± SD of three independent experiments. *P<0.05; **P<0.01.

Several reports have demonstrated that some constituents of *Gracilaria* possess anti-inflammatory properties [23], [33], [34]. COX-2, a pro-inflammatory enzyme, is linked to HCV-associated liver carcinogenesis [10]. To investigate whether AEGT can inhibit HCV-stimulated COX-2 expression, we analyzed the promoter activity, protein synthesis and enzyme activity of COX-2 in AEGT-treated Ava5 cells. As shown in Fig. 2A, HCV-stimulated COX-2 promoter activity was suppressed by AEGT in a concentration-dependent manner compared with 0.1% DMSO-treated Ava5 and parental Huh-7 cells (the fold of control), which was observed by a COX-2 promoter-linked luciferase reporter assay. These results indicated that AEGT down-regulated COX-2 expression at the mRNA transcription level. AEGT-induced COX-2 reduction was further confirmed by Western blot analysis (Fig. 2B). In addition, AEGT caused a concentration-dependent decrease in COX-2-mediated PGE_2_ biogenesis (Fig. 2C). Recently, many reports, including our previous studies, have demonstrated that the suppression of virus-induced COX-2 expression inhibits HCV replication [8], [14], [15], [17]. To examine whether the elimination of COX-2 expression was responsible for AEGT inhibition of HCV replication, we transiently overexpressed COX-2 in AEGT-treated Ava5 cells. Ava5 cells were transfected with a control vector or a pCMV-COX-2-Myc vector encoding the *cox-2* gene at increasing concentrations of transfected plasmid DNA (0.5, 1, 1.5 and 2 µg). Cells were incubated with AEGT (600 µg/ml), in which HCV-stimulated COX-2 expression and HCV replication were markedly blocked (Fig. 1 and Fig. 2). Western blot analysis revealed that AEGT-inhibited HCV NS5B protein synthesis (Fig. 3A, upper panel, lanes 3–6) was gradually attenuated by the increase in exogenous COX-2-Myc expression (middle panel) compared with the control transfected cells in the absence (lane 1) or presence of AEGT (lane 2). Consistent with previous results, qRT-PCR analysis revealed that exogenous COX-2-Myc augmentation significantly restored the AEGT-reduced HCV RNA levels in a concentration-dependent manner (Fig. 3B). Taken together, these findings suggest that COX-2 reduction was associated with AEGT anti-viral activity. NF-κB is a crucial transcription factor for COX-2 transactivation in response to viral infection and inflammation [17], [35]. To further elucidate whether the AEGT-mediated downregulation of COX-2 was modulated by NF-κB, we performed a luciferase assay specifically mediated via NF-κB activation. Ava5 and Huh-7 cells were transiently transfected with the *cis*-reporting plasmid pNF-κB-Luc and then incubated with or without AEGT for 3 days. As shown in Fig. 4A, increased NF-κB luciferase activity was significantly suppressed by AEGT in a concentration-dependent manner. Translocation of the NF-κB p65 subunit from the cytoplasm to the nucleus is required for NF-κB activation. We observed that compared with parental Huh-7 cells without AEGT treatment, the high level of virus-induced NF-κB p65 nuclear protein was gradually decreased to the basal level following AEGT treatment (Fig. 4B), suggesting that AEGT blocked HCV replication through the sustained suppression of NF-κB signalling pathways. We further performed the HCV JFH-1 infectious assay to confirm the anti-NF-κB activity of AEGT described above (Fig. 4C and D).

### AEGT Inhibits Gene Expression of Pro-inflammatory Mediators in HCV-infected and NS5A-expressing Cells

**Figure 5:**
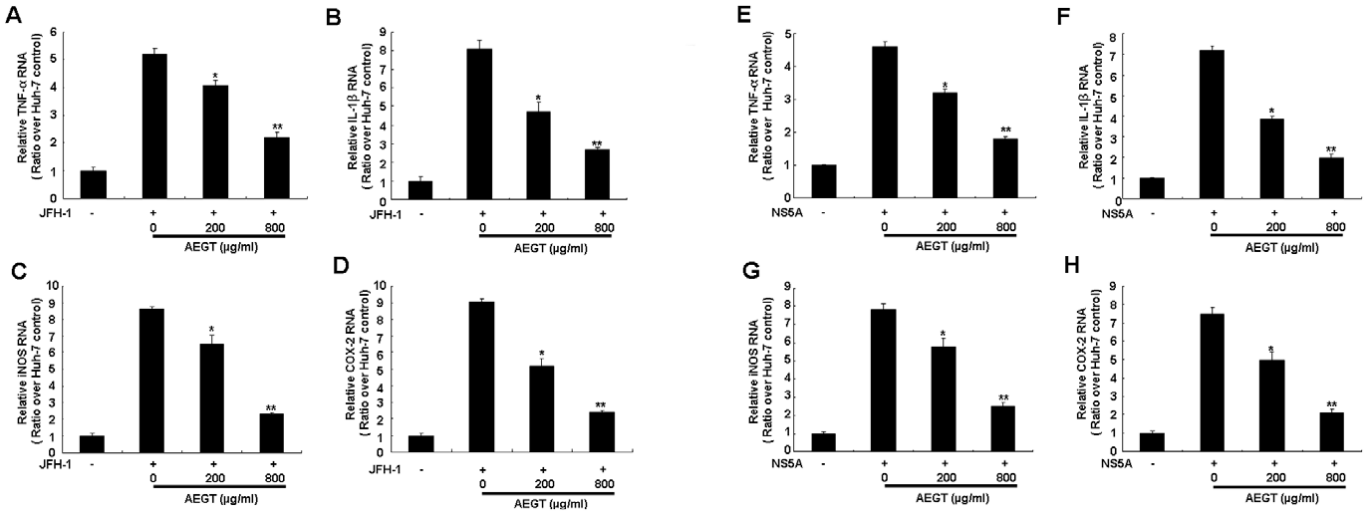
AEGT suppression of HCV-induced or NS5A-induced pro-inflammatory gene expression. HCV JFH-1-infected or pCMV-NS5A-Myc-transfected Huh-7 cells were incubated with indicated AEGT concentrations for 3 days. RNA levels of (A and E) TNF-α, (B and F) IL-1β, (C and G) iNOS and (D and H) COX-2 in each experiment were determined by qRT-PCR. The relative RNA level of each gene was normalized with cellular *gapdh* mRNA. Non-infected or non-transfected Huh-7 cells served as the basal control, which is defined as 1. Each value was represented as the mean ± SD of three independent experiments. *P<0.05; **P<0.01.

Chronic inflammation caused by HCV infection is considered as one of the major pathogenic mechanisms, A number of pro-inflammatory gene products as well cytokines, including COX-2, iNOS, TNF-α and IL-1β, are believed to play a critical role in inflammatory diseases [36], [37]. To examine the potential AEGT hepatoprotective actions against the HCV-stimulated inflammatory mediators described above, HCV JFH-1-infected Huh-7 cells were treated with different concentrations of AEGT for 3 days. qRT-PCR analysis demonstrated that compared to the uninfected cells, the elevated mRNA levels of these induced pro-inflammatory mediators were reduced in a concentration-dependent manner by AEGT (Fig. 5A–D). Among HCV proteins, NS5A is one of risk factors involving hepatocarcinogenesis through chronic inflammation [38], [39]. To further investigate the potential AEGT hepatoprotective actions against the NS5A-stimulated inflammatory mediators, NS5A-transfected Huh-7 cells were treated with different concentrations of AEGT for 3 days. Similar results for the reduction of NS5A-induced pro-inflammatory mediators by AEGT treatment were observed compared with the untreated cells (Fig. 5E–H).

## Discussion

In the present study, we showed that an active extract from *G. tenuistipitata* (AEGT) has potent inhibitory effects on HCV replication in both HCV subgenomic and infectious systems (Fig. 1). This is the first report of the potent anti-HCV activity from algal extract. Notably, combining AEGT with IFN-α synergistically inhibited HCV replication (Table 1), revealing that AEGT may be an effective therapeutic dietary alga in a combinational anti-HCV regimen. Studies on the mechanism of action of AEGT revealed that AEGT suppressed NF-κB-mediated COX-2 production at the transcriptional level (Fig. 2), and that this suppression revealed a critical mechanism underlying the attenuation of anti-HCV activity of AEGT by extraneous COX-2 overexpression in AEGT-treated HCV replicon cells (Fig. 3).

Recent advances in anti-HCV drug development have revealed many direct-acting antiviral agents (DAAs) that target HCV replication complexes, e.g. NS3/4A protease, NS5A and NS5B polymerase, at different stages of clinical development [40]. Due to the low fidelity of NS5B polymerase, increasing mutations throughout the viral genome usually reduces DAA susceptibility [41]. In this respect, cellular factors required for viral replication have emerged as promising drug targets because of lower mutation frequencies in the host genome than in the viral genome [42]. In addition, targeting the host factors provides an opportunity for the development of innovative drugs with broad-spectrum activity against all HCV genotypes. In addition to HCV infection, highly upregulated COX-2 levels during viral infection facilitated viral replication in other viruses such as cytomegalovirus [43], herpesvirus [44], respiratory syncytial virus (RSV) [45], enterovirus 71 [46] and West Nile virus [47]. In contrast, the elimination of virus-induced COX-2 expression by a selective inhibitor prevented the replication of other viruses such as human cytomegalovirus (HCMV) [48], H5N1 [49] and HCV [14], [16]. Thus, COX-2 may be a promising target for viral treatment. In the present study, AEGT efficiently blocked HCV replication by suppressing COX-2 expression (Fig. 3). Therefore, detailed investigation on COX-2 and its downstream signalling pathways involved in HCV replication are essential to develop an efficient therapeutic target against viral diseases.

An important HCC risk factor is chronic inflammation caused by HCV infection, in which HCV core and NS5A proteins act as etiologic proteins that greatly stimulate inflammatory mediators for the initiation and maintenance of cancer cell survival and growth [1]. NF-κB plays a primary role in inflammatory gene regulation upon viral infection. Moreover, increase in COX-2/PEG_2_ expression is linked to the progression of inflammation, which leads to carcinogenesis. Till date, little is known about the HCV regulatory pathways leading to COX-2 expression. Our results demonstrated that HCV dramatically induced NF-κB and COX-2 activation, whereas a gradual suppression of NF-κB- and COX-2-mediated transcriptional activity was observed upon AEGT treatment in HCV replicon cells, as depicted in Fig. 2 and Fig. 4. Based on these results, we suggested that the inhibitory effect of AEGT on numerous HCV-induced pro-inflammatory gene products is partly mediated via an NF-κB-dependent signalling pathway (Fig. 4). However, we cannot exclude the involvement of other cellular targets in the regulation of anti-HCV and anti-inflammatory activities of AEGT because this crude extract may possibly contain various active constitutes. Therefore, further purification of active AEGT constitutes is essential. Indeed, it is possible that crude extracts contribute to the synergistic actions through multiple targets against viral pathophysiological effects, although it is arduous to elucidate the detailed mechanisms. Further studies will be conducted to separate and identify active components for testing therapeutic protection against HCV-related diseases. In conclusion, the edible *G. tenuistipitata* extracts may be useful as a potential dietary supplement in the prevention and treatment of chronic HCV infection by simultaneous inhibition of viral replication, inflammation and carcinogenesis.
